# The Need of Better Water and Better Sewers

**Published:** 1908-07

**Authors:** 


### THE NEED OF BETTER WATER AND BETTER SEWERS.

The committee of twenty-five appointed by the Chamber of Commerce to investigate the demands for a $1,500,000 bond issue in Atlanta has made a very instructive report and one whose suggestions should be heeded.

It is shown that Atlanta has a much higher death rate than most other cities and that this is not due to climate or natural drainage, but to the following causes:

1. The use of surface closets and well water by over fifty thousand people, on account of insufficient sewerage and water mains.

2. The city water rendered impure at times by lack of proper filtration.

3. Clouds of filthy germ-laden dust blown into the face, eyes, ears and mouths of the people on the crowded streets.

The startling fact is also reported that one-third of the area and 40 per cent. of the population of Atlanta are without water mains or sewers. With 10,805 surface closets in the city and about 50,000 citizens drinking well-water, is it surprising that our annual mortality rate is high and that typhoid fever is such a common disease?

Instead of cutting down the proposed bond issue to $500,000, it should be increased to an amount sufficient to cover the cost of installing a modern and efficient sewerage system with drains for the street separate from those leading from the closets. The physicians of the city should unite in urging the necessity of a modern sewerage system and removal of all closets if our health is to be held at even an average.

